# The comparison of surgical patients with primary hepatic squamous cell carcinoma or adenosquamous carcinoma and surgical patients with hepatocellular carcinoma

**DOI:** 10.1186/s12957-015-0464-2

**Published:** 2015-02-28

**Authors:** Long Yan, Feng Xie, Cheng Yang, Lihan Yu, Tao Zheng, Jun Fu, Jiamei Yang

**Affiliations:** Department of Special Treatment and Liver Transplantation, Eastern Hepatobiliary Hospital, the Second Military Medical University, 255 Changhai Road, Shanghai, 200438 China

**Keywords:** Hepatic squamous cell carcinoma (SCC), Adenosquamous carcinoma (ASC), Hepatocellar carcinoma (HCC)

## Abstract

**Background:**

There are still several controversies and ambiguities in the aspects of primary hepatic squamous cell carcinoma and primary hepatic adenosquamous carcinoma. To further clarify the specific features of these two infrequent diseases and provide beneficial propose for clinical decision, we did this retrospective study.

**Methods:**

We reviewed the clinical features and outcomes of three primary hepatic squamous cell carcinoma (SCC) patients and six primary hepatic adenosquamous carcinoma (ASC) patients from January 1998 to December 2011 in Eastern Hepatobiliary Surgery Hospital in China. Meanwhile, 40 hepatocellular carcinoma (HCC) patients and 26 metastatic hepatic SCC or ASC patients who were hospitalized in the same period were also reviewed to make a comparison. In order to find out the definite survival information of SCC and ASC patients, 30 previous studies containing 37 primary hepatic SCC (16) and ASC (21) patients were reviewed, and their information of survival was analyzed together with the included patients in our hospital.

**Results:**

Serum tumor markers showed significant differences between primary hepatic SCC/ASC and HCC patients, especially for serum alpha fetal protein (AFP) level and carbohydrate antigen 19-9 (CA 19-9). On the pathologic performance, primary SCC/ASC tumor was rarely accompanied with tumor capsule. They presented peripheral hepatic lymph node metastasis more likely and showed low proportion of microvascular invasion. The median survival time of primary hepatic SCC/ASC patients after liver resection (LR) was 15 months. And the 1-, 3-, 5-year survival rates after LR were 60%, 24%, and 12%, respectively. Significant difference was not discovered when SCC and ASC patients were compared with HCC patients (*P* = 0.294). The median survival time after LR for primary SCC and ASC patients was respectively 23 months and 13 months.

**Conclusions:**

The comprehensive application of some clinical characteristics, histopathologic features, and imaging findings may be useful for us in making definite diagnoses for primary hepatic SCC and ASC patients preoperatively. And the treatment of liver resection was effective for those patients who met the selection criteria for liver resection.

## Background

Due to the prevalence of hepatitis and some other reasons, the morbidity of primary liver tumor is high in Asia, especially in China [1]. Though the majority of the primary tumors are hepatocellular carcinoma (HCC), some rare diseases also could not be ignored by the reason of their characteristics. Both primary hepatic squamous cell carcinoma (SCC) and adenosquamous carcinoma (ASC) are very rare. Few of them have been reported in the literatures. Primary hepatic SCC was considered to be associated with hepatic cysts [2-5], hepatolithiasis [6,7] Caroli’s disease [8], and others. Primary hepatic ASC was defined as a liver tumor containing definite components of both adenocarcinoma and squamous cell carcinoma, and sometimes it was considered as a variant of cholangiocarcinoma [9]. However, it was observed to have more aggressive clinical and pathological features than cholangiocarcinoma [10]. Both of SCC and ASC have squamous cell carcinoma components. These tumor components presented more malignant biological behavior, and that may be the common reason for the poor prognosis of the both [11].

We retrospectively investigated some primary hepatic SCC or ASC patients in our hospital to research the characteristics of these two kinds of diseases. Meanwhile, some patients in previous studies were also reviewed.

## Methods

### Patients

A total of 75 liver tumor patients, who had received liver resection (LR) during the period from January 1998 to December 2011 in Eastern Hepatobiliary Surgery Hospital, were picked out and included in this study.

There were just nine patients who suffered from histologically proven primary hepatic SCC (three patients) or ASC (six patients) had surgical treatment during the 14 years in our hospital, and all of them were included as the primary SCC/ASC group. Before their final diagnoses were made, each of these nine primary tumor patients had undergone imageological examination and hematological and blood biochemical determinations to preclude other primary tumor diseases. The HCC group contained 40 patients who were selected randomly from our data base. Considering the significant impact of liver cirrhosis to the prognosis of liver cancer patients and half of patients in the primary SCC/ASC group had liver cirrhosis (four patients), the HCC group patients were picked randomly according to the factor that nearly half of patients suffered cirrhosis and half did not. Twenty-six patients with metastatic hepatic SCC (16 patients) or ASC (10 patients) in the hospital during the same time composed the metastatic SCC/ASC group. The primary carcinomas of these patients contained lung cancer (12 patients, 46.2%), nasopharynx cancer (5 patients, 19.2%), gallbladder carcinoma (5 patients, 19.2%), esophagus cancer (3 patients, 11.5%), and vocal cord carcinoma (1 patients, 3.8%).

We only compared the metastatic SCC/ASC patients with the primary SCC/ASC patients on histopathologic findings. That was because in the clinical practice, it is difficult to determine whether the tumor is primary or metastatic by the postoperative histopathologic examination if other primary sites have not been discovered. So we intended to find some differences between them. All of the other comparisons, like demographic data, operative and laboratory findings, survival, were carried out between the primary SCC/ASC group and the HCC group.

### Clinical and laboratory data

The clinical and laboratory data of the primary SCC/ASC patients and HCC patients were reviewed retrospectively from the medical records and relevant databases of Eastern Hepatobiliary Surgery Hospital in China.

To analyze the clinical data, we reviewed the operation notes, like the number and size of tumors, TNM stage, and capsule of the tumors. The laboratory examinations were performed preoperatively, and the laboratory data contained biochemical tests, which reflected hepatic damage, such as prothrombin time (PT), total bilirubin (TBIL), serum alanine transaminase (ALT), and aspartate transaminase (AST). The data of serum alpha fetal protein (AFP), serum carbohydrate antigen 19-9 (CA 19-9), and serum carcinoembryonic antigen (CEA) before LR was analyzed at the same time. We also summarized the features of patients’ imagining and pathologic findings.

### Survival data

We retrospectively referred to the record databases and completed the follow-up work to get the data associated with survival. Forty-nine patients in the primary SCC/ASC group and the HCC group were followed up regularly until death or until the completion time of the data collecting work and writing the manuscript. The follow-up visit was conducted by phone.

As there were just nine primary hepatic SCC/ASC patients included in our study, in order to reduce the bias which might be brought by small sample, we reviewed some previous studies, and then synthetically analyzed the survival data they contained. We collected English papers and a few Chinese papers, which should have English abstracts, from Medline and Pubmed. The included studies should have the exact information of patients’ prognosis. All of the included patients should have no other tumor diseases and have accepted hepatic resections when their initial diagnosis had been made, and patients who died during the operation or few days after the operation would be excluded. Finally, 30 previous studies containing 37 primary hepatic SCC (16) and ASC (21) patients were included in our study to provide the survival information [12-33].

### Statistical analysis

The analyses were carried out by using SPSS 20. All data were presented as a median or the mean ± standard deviation (SD). The independent two-sample *t*-test was used to compare the numerical data. The Pearson chi-square test and Fisher’s exact test were performed for qualitative data. And the One-way analysis of variance (ANOVA), Kaplan-Meier, Cox regression analysis, was also used. A *P* value at <0.05 was regarded as statistically significant.

The study was approved by the Ethics Committee Eastern Hepatobiliary Surgery Hospital (approved number: EHBHKY-005-7), informed consent which has been conducted according to the principles expressedin the Declaration of Helsinki was obtained from each patient. All participantsprovided their written informed consent to participate in this study

## Results

### Demographic data and baseline characteristics

All related information of the primary SCC/ASC patients (9 patients) and primary HCC patients (40 patients) were summarized in Table 1 for the comparison of the demographic data and clinical characteristics. These two groups of patients shared almost the same gender and age composition. Of these patients, 7 (77.8%) men and 2 (22.2%) women were in the primary SCC/ASC group, and 33 (82.5%) men and 7 (17.5%) women were in the HCC group (*P* = 0.746). The average age was respectively 57.89 ± 11.062 and 54.55 ± 11.701 (*P* = 0.439). To maintain consistent baselines for all patients, the HCC group patients were selected according to the component ratio of patients with or without cirrhosis in the primary SCC/ASC group. So there were also no significant differences between these two groups of patients on the baseline liver function indexes, like TBIL, ALT, AST, and PT.

**Table 1:** **The demographic data and baseline characteristics of primary hepatic SCC**/**ASC patients and HCC patents**

	**Primary SCC/** **ASC group**	**HCC group**	***χ*** ^**2**^ **/F/** ***t***	***P***
Patients	9	40	-	-
Gender				
Male	7(77.8%)	33(82.5%)	0.105	0.746
Female	2(22.2%)	7(17.5%)		
Age (years)	57.89 ± 11.062	54.55 ± 11.701	0.781	0.439
Cirrhosis				
Yes	4(44.4%)	21(52.5%)	0.005	0.946
No	5(55.6%)	19(47.5%)		
AFP				
≤10 ng/ml	8(88.9%)	14(35%)	6.583	0.010
>10 ng/ml	1(11.1%)	26(65%)		
CA 19-9				
≤37 U/ml	4(44.4%)	39(97.5%)	14.625	0.000
>37 U/ml	5(55.6%)	1(2.5%)		
Prothrombin time (second)	12.24 ± 1.098	12.11 ± 1.153	0.288	0.775
TBIL				
≤2 mg/dl	8(88.9%)	27(67.5%)	0.766	0.382
>2 mg/dl	1(11.1%)	13(32.5%)		
ALT				
≤40 U/l	6(66.7%)	17(42.5%)	0.889	0.346
>40 U/l	3(33.3%)	23(57.5%)		
AST				
≤34 U/l	6(66.7%)	20(50%)	0.287	0.592
>34 U/l	3(33.3%)	20(50%)		
Extent of resection				
<Hemihep	7(77.8%)	35(87.5%)	0.051	0.821
≧Hemihep	2(22.2%)	5(12.5%)		
Transfusion				
Yes	7(77.8%)	23(57.5%)	0.562	0.454
No	2(22.2%)	17(42.5%)		
Postoperative TACE				
Yes	3(33.3%)	24(60%)	1.171	0.279
No	6(66.7%)	16(40%)		
Tumor size (cm)	8.33 ± 3.082	10.33 ± 6.645	−0.873	0.387
TNM stage				
I	1(11.1%)	0		
II	3(33.3%)	7(17.5%)		
III	1(11.1%)	26(65%)		
IV	4(44.4%)	7(17.5%)		

Serum tumor markers showed significant differences for these two groups patients due to the different tumor tissues. As a classical marker of HCC, serum AFP level appeared to have higher frequency of exceeding normal value in HCC patients (*P* = 0.01) and there was only one primary ASC patient (no primary SCC patient) who was detected with a high level of serum AFP. However, the performance of CA 19-9 was just the opposite. When we defined 37 U/ml was the highest normal level of serum CA 19-9, we found only one (2.5%) HCC patient exceeded this standard value, and the ratio was much less than primary SCC/ASC patients (*P* = 0.000).

Three (33.3%) patients in primary SCC/ASC group and 24 (60%) patients in HCC group adopted postoperative transhepatic arterial chemotherapy and embolization. When referring to the data of tumor sizes (the largest tumor size was chosen when several tumors were found in a patient), we found the average diameter of the tumors was generally large (>5 cm) for both two groups of patients (*P* = 0.387). When the primary liver tumors were staged by the TNM stage system [12], the primary SCC/ASC patients mainly distributed in stage II or IV, and the primary HCC patients mainly were stage IV. The extent of liver resection for most patients were less than hemihepatectomy (77.8% and 87.5%).

### Histopathologic findings

The comparison of general pathologic characteristics between primary SCC/ASC patients and HCC patients was listed in Table 2. From the result of statistical analysis, we found that, because the main component was squamous carcinoma, the liver tumor in primary SCC/ASC patients usually did not accompany tumor capsule. This feature could make the primary hepatic SCC/ASC show more malignant biological behavior. Though the difference was not significant (*P* = 0.191, >0.05), primary SCC/ASC patients presented peripheral hepatic lymph node metastasis more likely (4 patients, 44.4%). This performance might be related with these liver tumors’ malignant behavior. The microvascular invasion of tumors was quite different when comparing the pathology of the two groups of patients. The microvascular invasion only emerged in a few part of primary SCC/ASC (2 patients, 22.2%) but almost in all of the HCC patients (40 patients, 100%). The tumor tissues were staged in Edmondson-Steiner grading [13]. All these liver tumor patients were mainly distribute in stages II (moderately differentiated) and III (poorly differentiated). More than half of the primary SCC/ASC patients (6 patients, 66.7%) were stage II, and 75% of HCC patients (30 patients) were stage III. It seemed that tumor tissues in primary hepatic SCC and ASC were better differentiated than that in HCC.

**Table 2:** **The general pathologic characteristics of primary SCC**/**ASC patients and HCC patents**

	**Primary SCC/ASC group**	**HCC group**	***χ*** ^**2**^ **/F/** ***t***	***P***
Patients	9	40	-	-
Tumor capsule				
Yes	0	30(75%)	14.392	0.000
No	9(100%)	10(25%)		
Macrovascular invasion				
Yes	0	3(7.5%)	0.006	0.937
No	9(100%)	37(92.5%)		
Microvascular invasion				
Yes	2(22.2%)	40	30.222	0.000
No	7(77.8%)	0		
Lymph node metastasis				
Yes	4(44.4%)	7(17.5%)	1.712	0.191
No	5(55.6%)	33(82.5%)		
Histologic grade (Edmondson-Steiner)			4.060	0.044
I	0	0		
II	6(66.7%)	10(25%)		
III	3(33.3%)	30(75%)		

**Figure 1:**
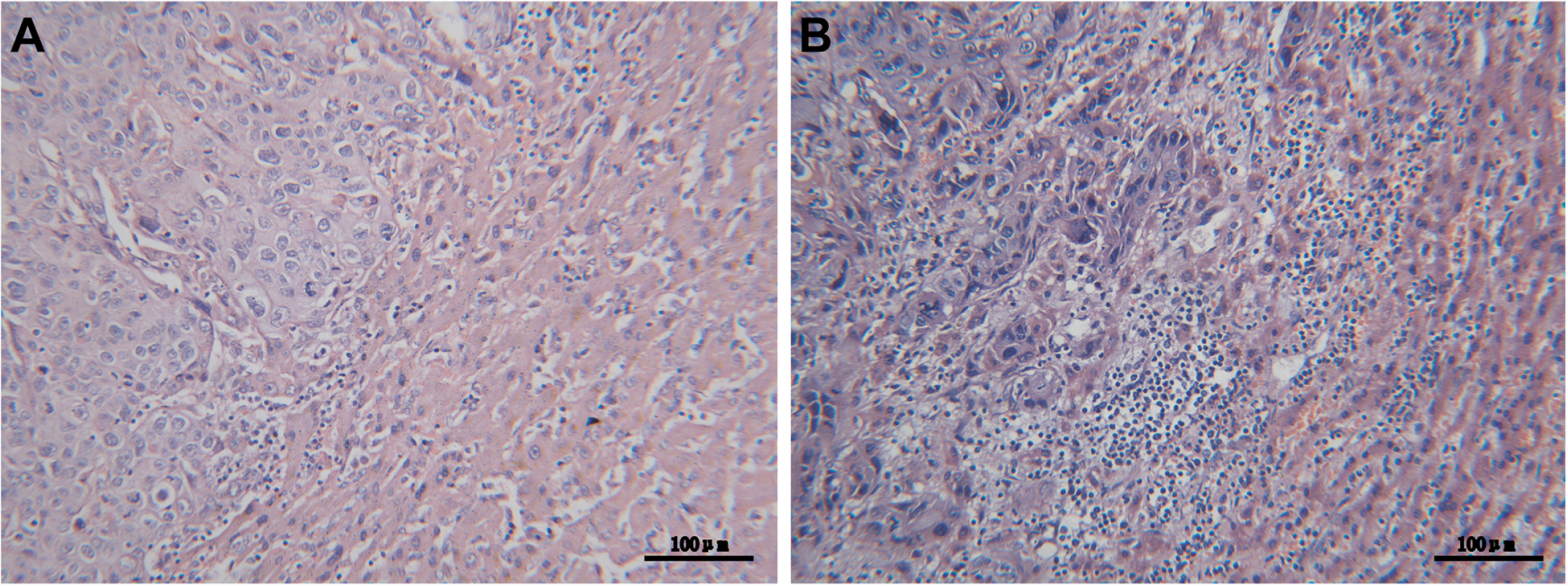
**Two typical HE tumor tissue slices.** **(A)** The squamous cancer cells were polygonal and arranged as strips, and their nucleuses were round or orbicular-ovate, most of which even had pathologic change, like karyomegaly and anachromasis (H & E stain, ×200). **(B** **)** Except the performance of squamous cell carcinoma components, adenocarcinoma cells were cubic or columnar and arranged like glandular tube (H & E stain, ×200).

Microscopically, the postoperative tumor tissue slice of primary hepatic SCC presented that the cancer cells were polygonal and arranged as strips, and their nucleuses were round or orbicular-ovate, most of which even had pathologic change, like karyomegaly and anachromasis. No capsule was found around the tumor and it appeared multifocal growth. Apart from the performance of squamous cancer cells, the tumor tissue of primary hepatic ASC arranged like glandular tube, adenocarcinoma cells were cubic or columnar and the tumor appeared infiltrative growth. We chose two typical HE tumor tissue slices from the nine primary carcinoma patients (Figure 1), of whom one was primary SCC (A), and the other was ASC (B).

In order to find the immunohistochemical characteristics of hepatic tumor pathology for primary SCC and ASC, we compared some immunohistochemical markers of tumor tissues between primary hepatic SCC/ASC and metastatic hepatic SCC/ASC (Table 3). Unlike the metastatic tumor group (80% positive CK 19), all of the primary group patients expressed positive CK 19, and a half of the primary hepatic ASC patients (three patients) even had strong positive expression. Considering that CK 19 generally appeared positive in the cholangiocellular carcinomas [34], the different performance of CK 19 between the four groups may provide some revelations for the origin of primary hepatic SCC and ASC. We found the percentage of positive CK 18 for primary SCC patients was larger than that of metastatic SCC (valid percent, 66.7%:42.9%), and that was also adapted to the comparison between primary ASC and metastatic ASC (valid percent, 83.3%:75%).

**Table 3:** **The immunohistochemical characteristics of primary SCC**/**ASC and metastatic SCC**/**ASC**

	**Primary SCC**	**Primary ASC**	**Metastatic SCC**	**Metastatic ASC**
Patients	3	6	16	10
CK 18				
Negative	1(33.3%)	1(16.7%)	8(57.1%)	2(25%)
Positive	2(66.7%)	5(83.3%)	6(42.9%)	6(75%)
CK 19				
Negative	0	0	3(20%)	2(20%)
Positive	3(100%)	6(100%)	12(80%)	8(80%)
CEA				
Negative	2(100%)	0	4(36.4%)	0
Positive	0	5(100%)	7(63.6%)	10(100%)

Two patients in primary hepatic SCC group (1 did not have the test) were negative for CEA, and in contrast to that, all primary hepatic ASC patients (5 patients) were positive for CEA, excepted 1 person who did not conduct the test.

### Survival

**Figure 2:**
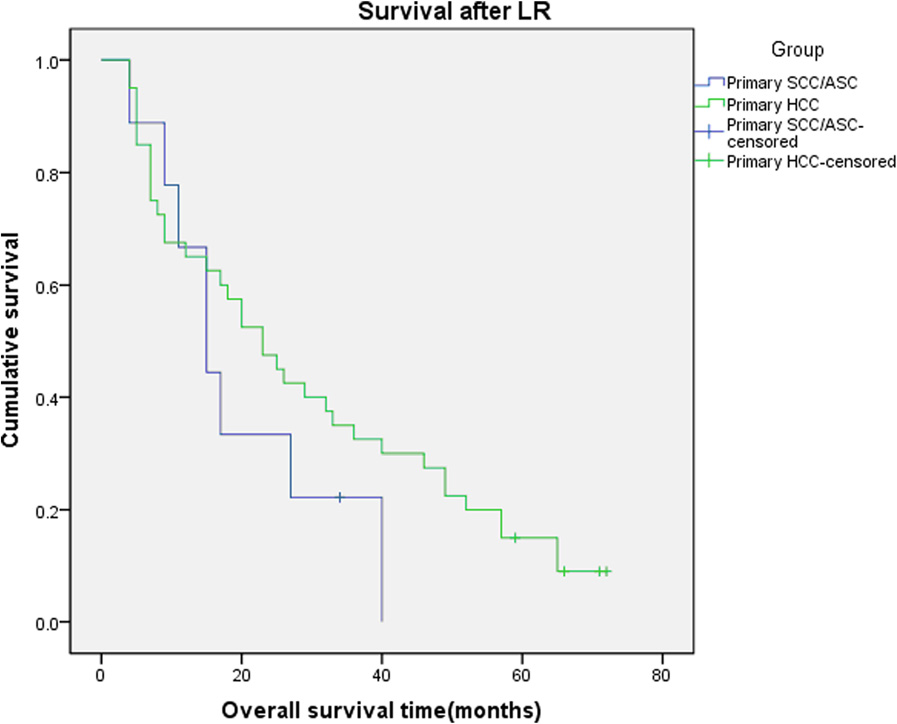
**Kaplan-** **Meier cumulative survival curves of the two groups of patients for overall survival** **(OS).**

As mentioned above, because the survival of the metastatic hepatic SCC and ASC patients might be significantly affected by the primary tumor disease, it was insufficient to analyze the survival just basing on the metastatic hepatic tumor diseases. So the survival analysis was only conducted in primary SCC/ASC and HCC patients (total 49 patients). The median time of follow-up was 20 months, arranged from 4 to 72 months. Till the end of follow-up period, there was only one patient alive in the primary SCC/ASC group. The median time of survival for these nine patients after LR was 15 months, and the mean of survival time was 19.778 ± 4.389 months. Primary SCC/ASC patients’ 1-, 3-, 5-year survival rates after LR were 67%, 20%, and 0%. For the 40 included HCC patients in this study, the median survival time after LR was 23 months and the mean of survival time was 29.405 ± 3.614 months. We also found their 1-, 3-, 5-year survival rates after LR were 68%, 35%, and 14%. The short-term survival after LR of these two groups of patients was nearly the same, but the long-term survival of the HCC patients seemed better than that of the primary SCC/ASC patients. However, we found no significant difference of the overall survival between two groups of patients (log rank *χ*^2^ = 1.216, *P* = 0.27, Figure 2).

**Figure 3:**
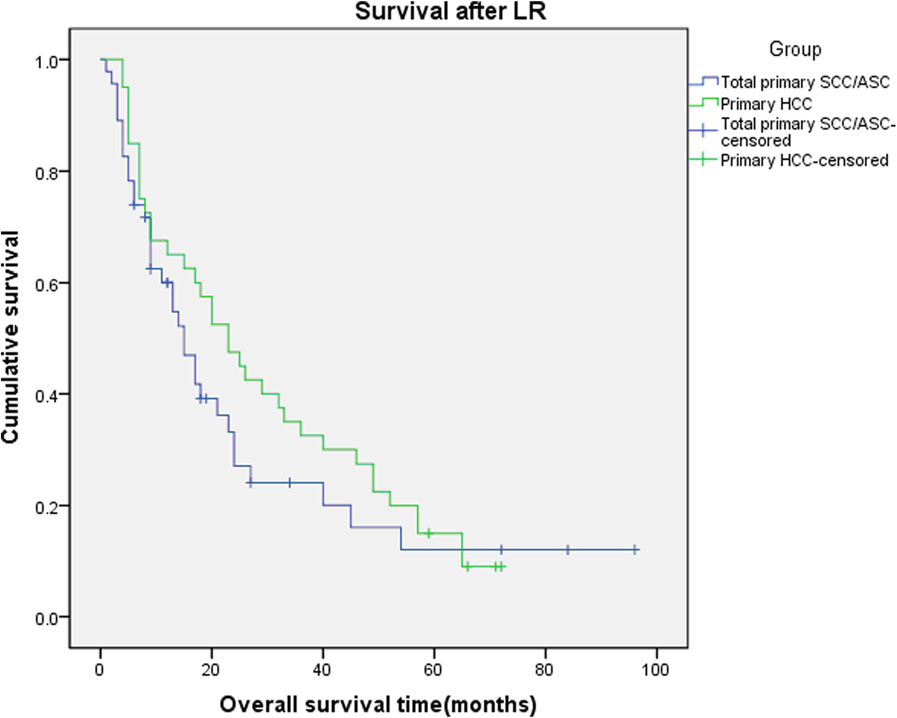
**Kaplan-** **Meier cumulative survival curves of the total primary SCC/** **ASC group** **(added 37 patients from previous studies)** **and HCC group of patients for overall survival** **(OS)** **after liver resection.**

**Figure 4:**
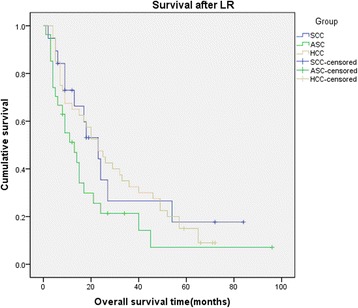
**Kaplan-** **Meier cumulative survival curves of primary SCC,** **ASC,** **and HCC group of patients for overall survival** **(OS)** **after liver resection.**

From the review of 30 previous studies, we extracted the survival information of 37 primary SCC (16, 43.2%) and ASC (21, 56.8%) patients, and their survival data was mixed together with our patients’ data to conduct the survival analysis. All of the 37 primary SCC/ASC patients from previous studies and our 9 primary SCC/ASC patients were defined as total primary SCC/ASC group (46 patients), which would be compared with those 40 HCC patients. The median survival time after LR of the total primary SCC/ASC patients was 15 months, which was the same with our included 9 SCC/ASC patients in our hospital. And the mean survival time after LR was 26.187 ± 4.876 months. The 1-, 3-, 5-year survival rates after LR for these primary SCC/ASC patients were 60%, 24%, 12%, respectively. We also did not find any significant difference when compared with HCC patients (log rank *χ*^2^ = 1.100, *P* = 0.294, Figure 3). At the same time, we compared all of primary SCC patients (19 patients) and primary ASC patients (27 patients) with HCC patients (40 patients), respectively. The median survival time after LR for primary SCC and ASC patients was 23 and 13 months. The 1-, 3-, 5-year survival rates after LR were 72%, 27%, and 18% and 51%, 21%, and 7% for SCC patients and ASC patients. Though the survival rates appeared to be different, from the analysis of comparison result, no significant difference was found when we compared the SCC and ASC patients with HCC patients respectively (log rank *χ*^2^ = 4.330, *P* = 0.115, Figure 4). This result was similar with what we obtained from the comparison of patients in our hospital. So from the statistical analysis results, we found that the survival after LR for primary hepatic SCC and ASC patients seemed similar with HCC patients.

## Discussion

Though various hypotheses have been set forth previously, the pathogenesis and precise steps leading up to the development of the carcinomas for both primary hepatic SCC and ASC are not exactly clear. Previous studies had reported that potentially pre-existent diseases, like hepatic cysts, containing congenital hepatic cyst or other benign non-parasitic cysts [2-5], calculus of intrahepatic duct [6], Caroli’s disease [7,8], may be the aetiological agents for primary hepatic SCC. In our primary SCC patients group, one patient had liver cirrhosis, and two had hepatic cyst, which was in accord with previous reports and might explain the pathogenesis. Similarly, though some pathogenesis of primary hepatic ASC had been proposed previously, it was still undefined. It was suggested in some previous studies that primary hepatic ASC might be caused by the squamous metaplasia of the intrahepatic bile duct [31]. The pathogenesis was also believed to be that the chronic inflammation continuously irritated the bile ducts or various congenital cysts of the biliary tracts [32], while someone proposed squamous metaplasia of denocarcinoma cells might be the etiologic factors [35]. In our study, the six included primary ASC patients contained one liver cirrhosis patients, one calculus of intrahepatic duct patients, and three hepatic cyst patients, which was also complied with those previous opinions about the pathogenesis of primary hepatic ASC.

Since the absence of sufficient evidence and undetermined pathogenesis for both primary hepatic SCC and ASC, most patients’ preoperative diagnosis could not be confirmed timely. A previous study reported that the level of serum squamous cell carcinoma-related antigen (SCC-Ag) might be a useful marker for preoperative diagnosis for ASC [36]. They found patients suffering from primary hepatic ASC might have a high level of serum SCC-Ag and this marker could also indicate tumor recurrence. In this paper, none of our primary hepatic SCC or ASC patients had definitive preoperative diagnosis.

We summarized and analyzed some kinds of preoperative clinical data and compared them between primary hepatic tumor (SCC and ASC) and HCC groups to explore appropriate methods for the diagnosis. Patients with primary hepatic SCC and ASC shared the similar performance of common serum tumor biomarkers, like negative level of AFP (≤10 ng/ml), and a part of positive level of CA 19-9 (>37 U/ml) that may distinguish them from most hepatocellular carcinoma and part of metastatic liver tumor, but it was incapable of picking them out from other kinds of metastatic liver tumor and most of usual intrahepatic cholangiocarcinoma. The imaging performance of primary hepatic SCC and ASC in our study did not have much unique characteristic. The CT images could tell a low density mass with irregular rim enhancement for primary hepatic SCC and ASC, which was similar to the presentations of intrahepatic cholangiocarcinoma, metastatic liver tumor, or even liver abscess. The imaging findings of primary SCC and ASC patients in our study were almost the same with previous reports, and they also found most of the MRI of primary hepatic SCC and ASC presented a low signal on T1WI image and high signal on T2WI image [33]. Someone detected that the ASC had a performance of high-signal T1WI image, which might relate to its solid-cystic structure and central necrosis [30,37].

Though a feasible and determinate preoperative diagnostic approach has not been established yet for primary hepatic SCC and ASC patients, the comprehensive application of testing tumor biomarkers, like AFP, CA19-9, and especially SCC-Ag, could be useful. At present, the definitive diagnosis is made on the basis of preoperative needle biopsy, or even has to rely on postoperative histological and immunohistochemical findings.

At present, both of the therapeutic regimes for primary hepatic SCC and ASC are not certain, due to the low incidence and belated definitive diagnosis. Surgical procedures may be a more important treatment for both two [6-11,25-33]. Besides the resection, previously reported methods included transcatheter arterial infusion chemotherapy (TACE), which was considered to be useful for both primary hepatic SCC and ASC patients [22]. Systemic chemotherapy was also reported in the therapeutic course of SCC and ASC [21,33]. Although these therapeutic methods were reported to have significant treatment effects, they usually were adopted just as adjuvant therapy [29]. In previous studies, whether TACE or systemic chemotherapy was always undertaken before or post resections.

With the high malignancy of tumor, most of the primary SCC and ASC patients had dismal prognosis [38]. The squamous cell carcinoma components always present more malignant biological behavior, and it may be the common reason for the poor prognosis of these two diseases [39]. Takahashi found that, even in primary adenosquamous carcinoma of liver, the squamous cell carcinoma components had highly proliferative activity [9]. But if surgery resection was undertaken, the situation might be improved. From the analysis of the survival data of the included patients in our hospital and the patients in the previous studies, we even did not find any significant difference between the primary SCC/ASC patients and the HCC patients about the survival after the treatment of LR (log rank, *P* > 0.05). Of course, maybe we can not be so optimistic because we could not ignore some reasons which may play roles in achieving this improvement. Because of the high malignancy of primary hepatic SCC and ASC, the tumor progresses rapidly, and it certainly leads to a small number of patients could get the treatment of LR according to the guideline. The prognoses of the rest were gloomy and we could not get sufficient information of this crowd who did not receive surgical treatment yet.

## Conclusions

Though it was difficult to make a definite diagnosis for primary hepatic SCC and ASC patients preoperatively in the condition that biopsy has not been conducted, there still were some features for them in clinical characteristics, histopathologic features, and imaging findings. And the treatment of liver resection was effective for some of these patients. The low incidence of primary hepatic SCC and ASC resulted in difficulties to carry out large sample studies. Multicenter studies could be performed to further clarify the specific features and to confirm therapeutic methods.
